# Detection of Nipah virus in *Pteropus medius* in 2019 outbreak from Ernakulam district, Kerala, India

**DOI:** 10.1186/s12879-021-05865-7

**Published:** 2021-02-09

**Authors:** A. B. Sudeep, Pragya D. Yadav, Mangesh D. Gokhale, R. Balasubramanian, Nivedita Gupta, Anita Shete, Rajlaxmi Jain, Savita Patil, Rima R. Sahay, Dimpal A. Nyayanit, Sanjay Gopale, Prachi G. Pardeshi, Triparna D. Majumdar, Dilip R. Patil, A. P. Sugunan, Devendra T. Mourya

**Affiliations:** 1 ICMR-National Institute of Virology, 20-A, Dr. Ambedkar Road, Pune, Maharashtra 411001 India; 2 Maximum Containment Laboratory, Indian Council of Medical Research-National Institute of Virology, Sus Road, Pashan, Pune, 411 021 India; 3 ICMR-National Institute of Virology, Kerala unit, Alappuzha, India; 4 Indian Council of Medical Research, Ansari Nagar, New Delhi, India

**Keywords:** Ernakulum, Nipah virus, NGS, IgG ELISA, *Pteropus spp*

## Abstract

**Background:**

In June 2019, Nipah virus (NiV) infection was detected in a 21-year-old male (index case) of Ernakulum, Kerala, India. This study was undertaken to determine if NiV was in circulation in *Pteropus* species (*spp)* in those areas where the index case had visit history in 1 month.

**Methods:**

Specialized techniques were used to trap the *Pteropus medius* bats (random sampling) in the vicinity of the index case area. Throat and rectal swabs samples of 141 bats along with visceral organs of 92 bats were collected to detect the presence of NiV by real-time reverse transcriptase-polymerase chain reaction (qRTPCR). Serum samples of 52 bats were tested for anti-NiV Immunoglobulin (Ig) G antibodies by Enzyme-Linked Immunosorbent Assay (ELISA). The complete genome of NiV was sequenced by next-generation sequencing (NGS) from the tissues and swab samples of bats.

**Results:**

One rectal swab sample and three bats visceral organs were found positive for the NiV. Interestingly, 20.68% (12/58) of *Pteropus* were positive for anti-NiV IgG antibodies. NiV sequences of 18,172; 17,200 and 15,100 nucleotide bps could be retrieved from three *Pteropus* bats.

**Conclusion:**

A distinct cluster of NiV sequences, with significant net-evolutionary nucleotide divergence, was obtained, suggesting the circulation of new genotype (I-India) in South India. NiV Positivity in *Pteropus* spp. of bats revealed that NiV is circulating in many districts of Kerala state, and active surveillance of NiV should be immediately set up to know the hotspot area for NiV infection.

**Supplementary Information:**

The online version contains supplementary material available at 10.1186/s12879-021-05865-7.

## Background

**Fig. 1:**
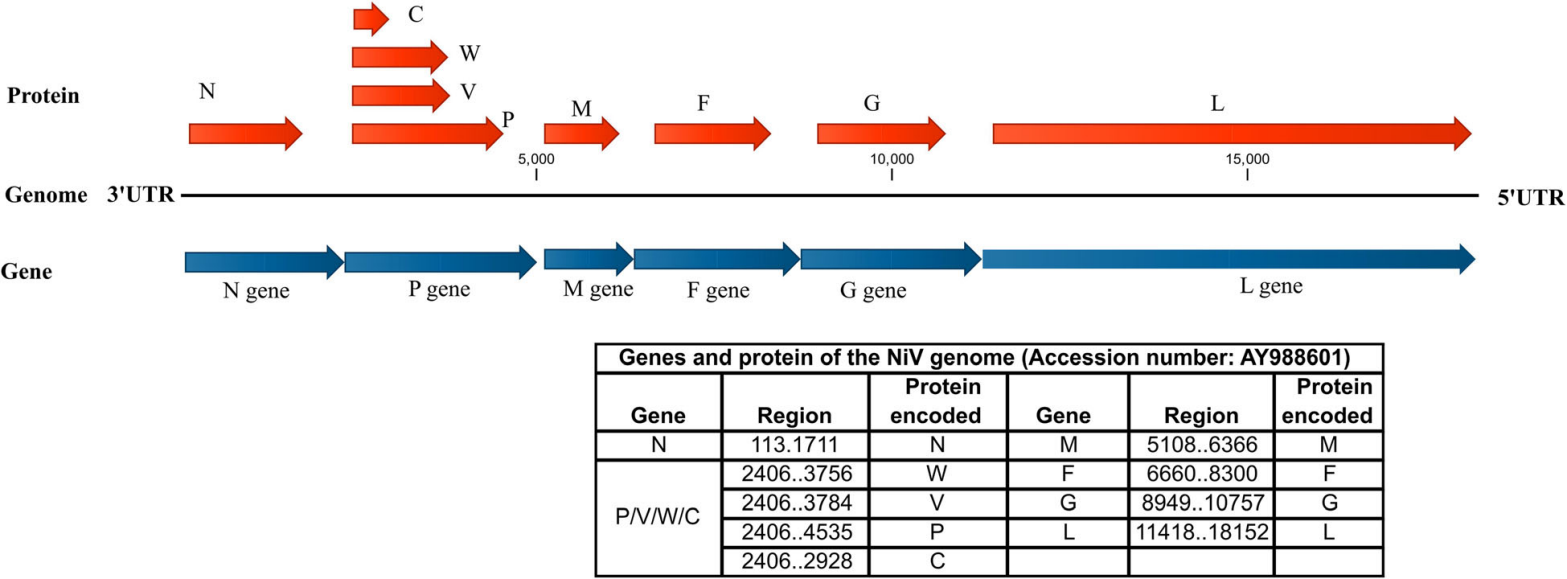
Nipah virus genome. Linear diagrammatic view of the different genes present on the NiV genome along with the proteins encoded by them. The figure was obtained from CLC Genomics Workbench v 11 and modifed for better vizualization

Nipah virus (NiV), belonging to genus *Henipavirus* (family *Paramyxoviridae*), caused high mortality in humans and was first reported from Malaysia and Singapore during 1998–99 [1, 2]. The Nipah virus genes and the protein encoded by them are depicted in Fig. 1. The virus caused severe febrile encephalitis-like symptoms in pig handlers with a case fatality rate (CFR) of approximately 40% in Malaysia, whereas, in Singapore, one fatal case was reported among 11 infected persons [1, 2]. Subsequent studies have shown *Pteropus spps*. as the reservoir for NiV [1]. These bats are widespread in South Asia as well as Northern Australia [3]. The presence of NiV has been reported from neighboring countries of India viz. Singapore, Malaysia, and Bangladesh, with a mortality rate ranging from 40 to 70% [4]. In India, the first outbreak was reported in the Siliguri district in 2001, followed by Nadia district in 2007, in West Bengal state, which shares a border with Bangladesh [5, 6]. In 2018, the NiV outbreak was reported in Kozhikode, Kerala, with a CFR of approximately 89% [7, 8]. It was found that *Pteropus* spp. of bats were the probable source of NiV infection in the 2018 outbreak in Kerala [8].

During the present outbreak, a 21-year-old male student was admitted to a private hospital of Ernakulam district, Kerala state, with persistent fever for 10 days, followed by acute encephalitic symptoms for 2 days. The clinical samples, including blood, serum, urine, and cerebrospinal fluid (CSF) were referred to the Indian Council of Medical Research (ICMR)-National Institute of Virology (NIV), Pune to rule out NiV infection in the case. Nipah viral RNA was detected from CSF and urine samples by real-time reverse transcriptase-polymerase chain reaction (qRT-PCR). Anti-Immunoglobulin (Ig) M antibodies were detected in serum samples by Enzyme-Linked Immunosorbent Assay (ELISA) [7, 8].

The present study was undertaken to ascertain the NiV virus circulation in *Pteropus* and *Rousettus* bats in those areas where index case visited in the near month to understand the probable exposure. Bats were trapped and screened for NiV RNA and anti-NiV IgG antibodies.

## Methods

### Ethics statement

The study was approved by the Institutional Animal Ethics Committee (IAEC) of ICMR-NIV, Pune (IAEC/2019/MEZ/04). The permission from Principal Chief Conservator of Forest, Kerala state was obtained for trapping of bats and performing this study.

### Study area

**Fig. 2:**
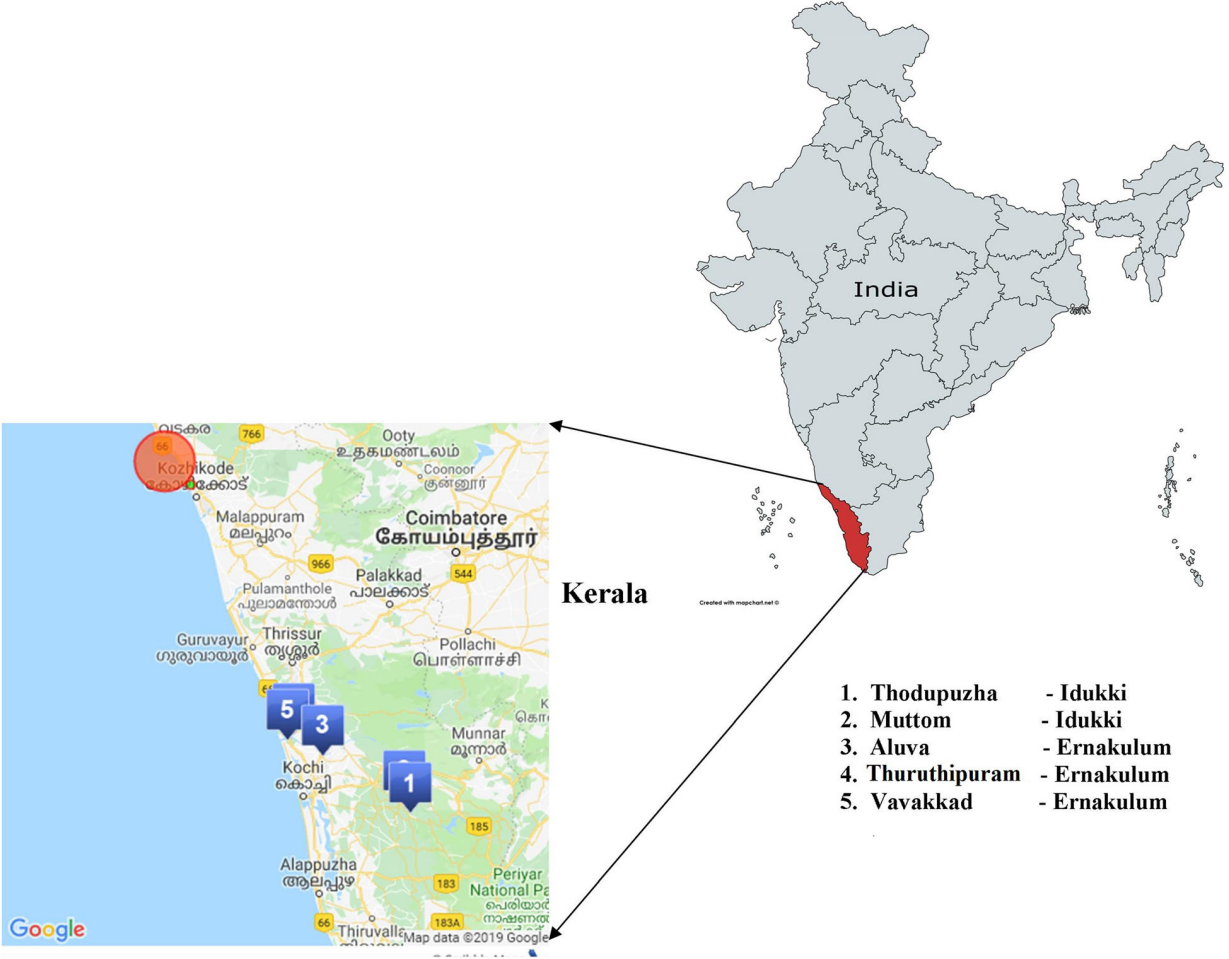
Five sites of bat collection in Kerala state during NiV outbreak, June 2019. Footnotes: The outline of the Indian map was obtained from https://mapchart.net/india.html. The Google details were designed by authors themselves using Scribble map available from https://www.scribblemaps.com. Authors have created the figure themselves, using the links mentioned above

Bats were captured from the five-kilometer radius of the index case residence (Ernakulum district) and the college area (Idukki district) between 5th and 10th day after detection of index case. The five sites from where the bats were trapped were: Vavakkad, Aluva, Thuruthipuram in Ernakulam district and Thodupuzha, Muttam in Idukki district as per the proximity to the residence and college (Fig. 2).

The selected sites except for Thuruthipuram had bat roosts with numbers ranging from a hundred to several thousand. Thuruthipuram was chosen due to the presence of guava and almond tree plantations, frequently visited by bats, which were just 100–200 m away from the residence of the index case. Vavakkad site was within 5 km from the index case house and had a permanent roost with approximately 200 bats. Thodupuzha site was within a 5 km radius from the college, where the index case was studying and had a massive roost (nearly 10–20 thousand bats), spread over about half an acre of land. Muttam was towards the Idukki dam area and was one of the oldest roosts in the area spread over an acre of land with approximately 50,000 bats.

### Trapping of bats and collection of samples

Bats [*Pteropus medius* (109) and *Rousettus leschenaultii* (32)] were trapped randomly using mist nests hoisted on aluminum inter-connectible poles at the height of 48 to 56 ft. The nets were set up to trap the bats between 6 and 7 PM before the foraging activities of the bats were initiated. The trapped bats were collected in the early morning (4 to 6 am) and were anesthetized with isoflurane inhalation. Throat and rectal swabs specimens were collected from 141 bats in virus transport medium (HiViral™ Transport Medium, HIMEDIA) and stored immediately in dry ice. The blood samples (around 2–3 ml) were collected from the wing (cephalic) vein of 78 bats [*Pteropus spp* (*n* = 58) and *Rousettus spp* (*n* = 32)] and serum was separated. After recovery from anesthesia, the bats were released back [7, 8]. All the samples (serum, throat, and rectal swabs) were transported to ICMR-NIV, Pune in dry ice (Table 1). Ninety-two bats from five sites were euthanized with Isoflurane inhalation overdose. They were transported to ICMR-NIV, Pune, in liquid nitrogen to check for the presence of the NiV RNA in visceral organs. Necropsy was performed on 92 captured bats taking all biosafety precautions in the Biosafety Level (BSL)-4 laboratory at ICMR-NIV, Pune. The organs (brain, heart, lung, liver, spleen, intestine, kidney, and reproductive organs) were collected aseptically. These organs were then triturated in minimum essential medium (MEM) (Life Technologies Corporation, USA), and tissue homogenate was stored at − 80^0^ C for further use.

**Table 1:** Details of bat samples collected from five sites during the NiV outbreak in Ernakulum district, Kerala state, India in 2019

Location/Site of the collection in Kerala state	Bat species	NiV positive samples / total number of tested samples [by Real-Time RT PCR]			NiV positive samples/ total serum samples tested [by anti-NiV IgG ELISA]
		Throat swab	Rectal swab	Visceral organs	Serum
Site 1-Near River Thodupuzha, Idukki district	*Pteropus medius*	0/54	1/54^a^	2/25^b^	4/23
Site 2-Muttom, Thodupuzha, Idukki district	*Pteropus medius*	0/8	0/8	0/8	0/8
Site 3 -Aluva, Ernakulum district	*Pteropus medius*	0/29	0/29	1/20^c^	6/19
Site 3-Aluva, Ernakulum district	*Rousettus*	0/32	0/32	0/31	0/20
Site 4-Thuruthipuram, Thekekkara Panchayat, Ernakulum district	*Pteropus medius*	0/4	0/4	0/4	1/4
Site 5-Vavakkad, Paravoor, Thekekkara Panchayat, Ernakulum district	*Pteropus medius*	0/14	0/14	0/4	1/4
**Total number of bats specimens positive/ tested**		**0/141**	**1/141**	**3/92**	**12/78**

^a^Rectal swab positivity for NiV RNA (Ct-36.51) trapped from site-1 was observed in only one *Pteropus* bat [MCL-19-bat-572], which was also positive for NiV RNA by qRTPCR in liver/spleen (Ct-26.47), kidney (Ct-27.9), lung (Ct-26.49), heart (Ct-30.83), reproductive organ (Ct-33.25), and intestine (Ct-31.65)

^b^ Another *Pteropus* bat [MCL-19-bat-574] from site 1 were also positive for presence NiV RNA by Real-time RT-PCR in liver/spleen (Ct-28.96), kidney (Ct-34.14), brain (Ct-33.92), lungs (Ct-32.68), heart (Ct-33.58); however, the serum samples of these bats were negative for the presence of anti-NiV IgG antibodies

^c^ One *Pteropus* bat [MCL-19-bat-618] from site 3 was positive for the presence of NiV RNA by Real-time RT-PCR in liver/spleen (Ct- 33.62) and for anti-NiV IgG antibodies in serum

### Detection of NiV RNA by qRTPCR

RNA was extracted from the throat, rectal swabs and tissue homogenates using the Magmax RNA extraction kit (Applied Biosystems, USA) as per manufacturer’s instructions. NiV-specificqRT-PCR was performed as described earlier [9].

#### Detection of anti-NiV bat IgG antibodies by ELISA

The bat serum samples were heat-inactivated (56^0^ C for 30 min). ELISA plates were coated overnight at 4 °C with NiV infected *Vero CCL81* cell lysate antigen in 1:20 dilution, and uninfected *Vero CCL81* cell lysate was used as a negative antigen. A volume of 100 (micro-liter) μl (1:100 dilution) bat sera samples were added and incubated for 1 hour at 37 °C. 100 μl of anti-bat IgG horseradish peroxidase (HRP) conjugate (Thermo fisher scientific) in 1:2000 dilution was added and incubated for 1 hour at 37 °C. 3,3′,5,5′-Tetramethylbenzidine (TMB) (Cat No29994, Clinical Science Product incorporation NeA blue, USA) was added and incubated at 37 °C for 12–15 min. The reaction was stopped by adding 1 normal (N) Sulfuric acid (H_2_SO_4_), and plates were read at 450 nm (nm). The plates were washed four times using 10 millimolar (mM) phosphate buffer saline (PBS) pH 7.4 with 0.1% Tween-20 (Sigma, USA) at the end of each step. Positive and negative controls available at ICMR-NIV, Pune were included in the test. For sensitivity and specificity, indigenously developed ELISA was compared with anti-Nipah bat IgG ELISA reagents provided by the Center for Disease Control and Prevention (CDC), Atlanta USA. The sensitivity and specificity of indigenous anti-NiV bat IgG ELISA were 100.0 and 83.3%, respectively, in comparison to ELISA developed by CDC reagents, which is considered as the gold standard [10].

### Next-generation sequencing (NGS) and phylogenetic analysis of NiV positive bat specimens

Efforts were made for the amplification of bat specimens using the PCR multiplexing method. The multiplexing reaction was performed with the primer pools designed using the Primal Schema tool http://primal.zibraproject.org/ using Kerala NiV sequences MH523640 [11]. The primers designed for the complete NiV sequencing were of 400 base pairs (bps) with an overlap of 75 bps. The steps involved are as follows: 5 μl of the RNA (liver, spleen, kidney, lung, and rectal swab) was amplified using 35 cycles of PCR. The amplified PCR product was loaded on 1.5% agarose gel and electrophoresed. The PCR products were purified using the Qiagen gel extraction kit (Qiagen, Germany). The amplicon generated from this extracted product was used from the second strand synthesis step, as described in the TruSeq Stranded mRNA LT Library preparation kit (Illumina, USA). This purified amplicon was then used further for library preparation as described in the TruSeq Stranded mRNA LT Library preparation kit (Illumina, USA), beginning at the second strand synthesis step as described earlier [12]. The generated reads were analyzed using CLC Genomics workbench version 11.0. Reference-based mapping was used to retrieve the genomic sequence of the NiV. Reference NiV sequences were downloaded from the GenBank database and were aligned using MEGA software version 7 [13]. An evolutionary tree was generated using the best-predicted model for the N gene of the Nipah virus. A bootstrap replication of 1000 cycles were performed to assess statistical robustness.

## Results

### Detection of NiV RNA in the bat samples

One (MCL-19-bat-572 *Pteropus* bat; collected from site-1 Thodupuzha, Idukki district) out of the total 141 rectal swabs collected was positive for NiV RNA by qRT-PCR while none of the throat swabs [*n* = 141] samples were positive. The visceral organs, including liver/spleen, kidney, lung, heart, reproductive organ, and intestine of MCL-19-bat-572 showed NiV RNA positivity [Cycle threshold (Ct) values ranged from 26.47 to 36.51]. MCL-19-bat-574 also collected from site-1 showed NiV positivity in liver/spleen, kidney, brain, lung, heart [Ct values ranged from 28.96 to 34.14]. MCL-19-bat-618 *Pteropus* bat trapped from site-3 (Aluva, Ernakulam district) showed positivity in Liver/spleen [Ct value 33.62] (Table 1).

### Detection of anti-NiV IgG antibodies in bat serum samples

Anti NiV-IgG bat antibodies were detected in 12/58 (20.68% positivity) *Pteropus* bat sera from sites 1,3,4 and 5, whereas none of the *Rousettus* sera were found positive (Table 1).

### NGS and phylogenetic analysis of NiV positive bat specimens

**Fig. 3:**
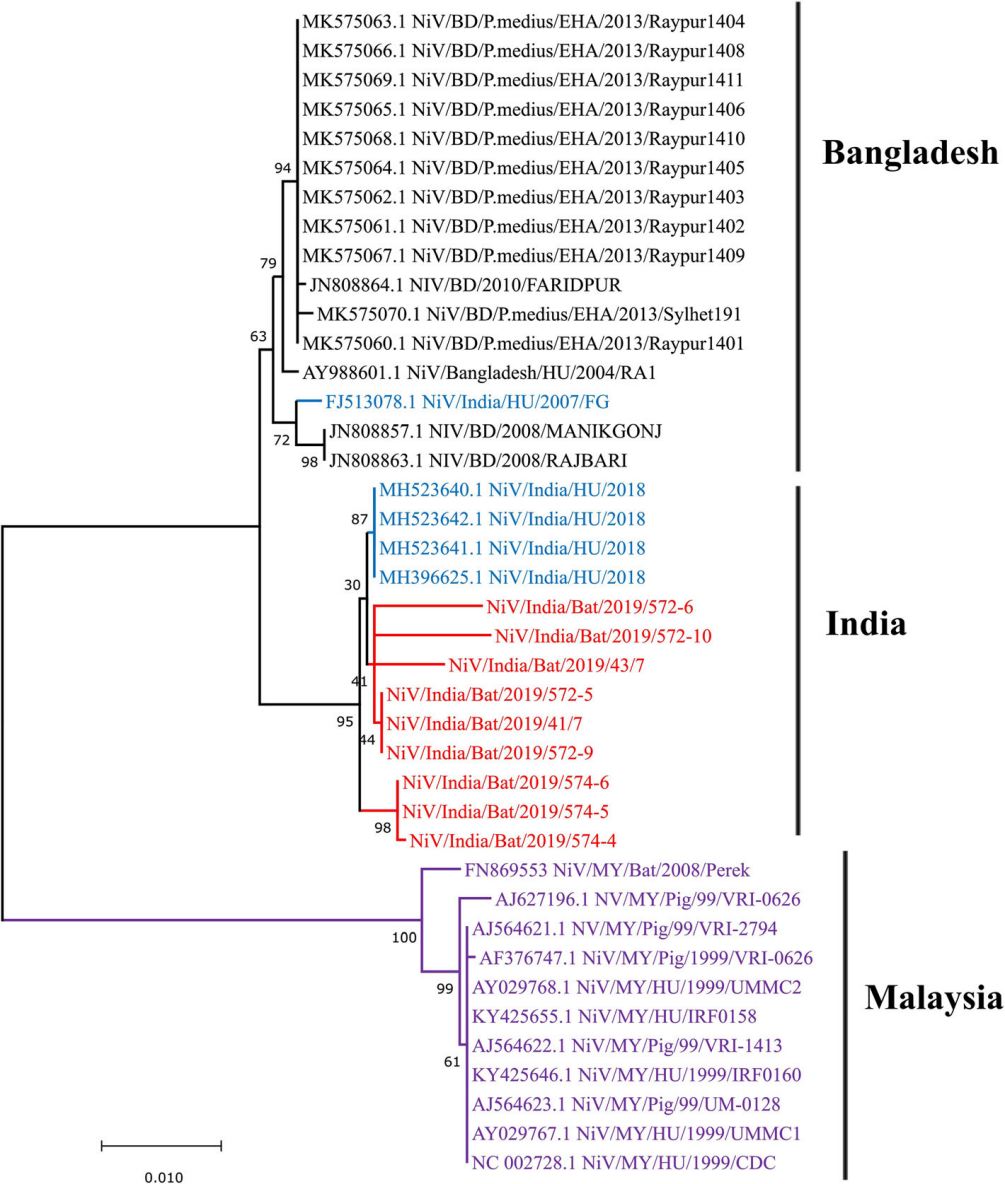
Maximum-Likelihood tree for the N gene of the Nipah virus sequence from Kerala, India, 2019, with other NiV reference sequences. Bootstrap replication of the 1000-replication cycle was used for the statistical assessment of the generated tree. Scale bars indicate nucleotide substitutions per site. Color code for the tree: Black- NiV sequences from Bangladesh; Purple – Malaysian NiV sequences; Blue –Indian NiV sequences; and Red color is for the Niv sequences from India retrieved in this study

The complete genome of the NiV (18,172 nucleotide bps) could be retrieved from the kidney sample of one *Pteropus* bat, using the reference-based mapping approach [9]. NiV sequences could also be retrieved from the liver/spleen sample of the two *Pteropus* bats with a length of approximately 17,200 and 15,100 bps. The details of the NiV genomic regions retrieved from the bat samples are given in Supplementary Figure 1. The evolutionary tree demonstrated a distinct branch for the Indian NiV sequences (Fig. 3). NiV human sequences from Kozhikode, Kerala 2018 outbreak, and the NiV bat sequences from the current 2019 study area branched out from the Bangladesh sequences. The NiV strains circulating in the southern region of India are distinct from the B genotype and formed a separate cluster. The net-evolutionary nucleotide divergence found between the NiV strains from South India (Kerala) to Bangladesh and Malaysia strains is 1.96 and 8.24%, respectively. Gene-wise nucleotide and amino acid net-evolutionary divergence for Malaysia and Bangladesh strains concerning Indian sequences are given in Table 2. Bangladesh and West Bengal sequences showed a divergence of 0.75%, while Kerala and Bangladesh sequences showed the divergence of 2.2%. Hence we hypothesize the existence of two types of genotypic sequences in India and propose a separate genotype for Kerala, Indian strains as ‘I-India’ genotype. The percent nucleotide divergence for the sequences used in the study and the amino acids changes for the representative sequences are given as Supplementary files 1, and 2 respectively. The pairwise comparison for each NiV gene with respect to its reference Bangladesh strain is given in supplementary Table 3.

**Table 2:** Gene-wise percent nucleotide and amino acid net-evolutionary divergence for Malaysia and Bangladesh NiV strains for Indian NiV sequences

Genes	N		P		M		F		G		L	
	NA^a^	AA^a^	NA^a^	AA^a^	NA^a^	AA^a^	NA^a^	AA^a^	NA^a^	AA^a^	NA^a^	AA^a^
Bangladesh-West Bengal, India	0.5	0.2	0.9	0.9	0.2	0.3	0.4	0.1	0.5	0.3	0.6	0.2
Bangladesh-Kerala, India	1.1	0.6	2.0	1.9	1.0	0.0	1.5	0.2	1.9	1.1	1.6	0.3
West Bengal, Kerala, India	1.4	0.7	2.0	2.1	1.2	0.3	1.7	0.4	2.0	1.2	1.5	0.1
Malaysia-Bangladesh	5.5	1.4	8.2	8.0	6.5	1.0	6.2	1.1	6.9	4.3	6.5	1.6
Malaysia- West Bengal, India	5.9	1.6	8.2	8.1	6.6	1.3	6.4	1.2	7.0	4.3	6.4	1.5
Malaysia-Kerala, India	6.0	1.6	8.2	8.8	6.8	1.0	6.5	1.3	7.1	4.1	6.6	1.4

^a^*NA* nucleotide, *AA* Amino acid

## Discussion

India has once again garnered the world’s attention with the outbreak of NiV in Ernakulum district of Kerala, within a year of an outbreak in Kozhikode district [8, 9]. Even though the mode of transmission of NiV to humans was well studied in Malaysia, Singapore, Philippines and Bangladesh outbreaks, the transmission dynamics in recent outbreaks in India in 2018 and 2019 needs to be further studied. Bat NiV positivity from within 5 km radius of the residence and college of the index case probably explains the role of *Pteropus spps*. in NiV transmission. The rectal swab, liver/spleen, lung, kidneys, intestine, and reproductive organ in *Pteropus medius* collected from Thodupuzha, possibly demonstrated bat as a potential source of NiV infection to the index case although, the route of transmission remains unclear. More intensive studies are needed to understand the dynamics of NiV transmission.

The mode of transmission of NiV differed at each outbreak and has shown the presence of an intermediate host in Malaysia, Singapore and Philippines [2, 14]. However, in India and Bangladesh, no intermediate host could be determined even though pigs in Bangladesh were experimentally shown to shed the virus in body fluids and excreta [15, 16]. The *Pteropus spps* were also identified as the most likely source of NiV transmission during the 2018 outbreak in Kozhikode [8, 9]. Since no effective drugs or prophylaxis are available for NiV infection, preparedness for early diagnosis is essential. The timely detection and confirmation of the etiological agent coupled with the lessons learned from the 2018 Nipah virus outbreak in the Kerala state have helped in early isolation of the patient, further preventing human to human transmission.

No NiV positivity could be detected in 32 *Rousettus spps*, sampled from the 2019 and 2018 Kerala outbreak area, indicating *Rousettus* spp. may not have a role in the NiV transmission cycle. The presence of anti-NiV IgG antibodies in 20.68% of the *P. medius* suggests NiV seropositivity in the current outbreak region. Four sampling sites showing the presence of anti-NiV IgG bat antibodies warrants active human surveillance for early detection of NiV cases.

Further, the presence of distinct clusters in the sequence analysis from the outbreaks in southern India (Kozhikode and Ernakulum districts of Kerala state in humans and bats) guides us to hypothesize the presence of a new ‘Indian (I)’ genotype circulating in India.

## Conclusions

NiV positivity in *Pteropus medius* during the current outbreak (2019), suggest the probable role of bats in NiV transmission in Ernakulam, Kerala state. Authors would like to put forward the proposal for the circulation of new NiV strain “India (I)” in the Southern part of India, which is different from Bangladesh and Indian northeast NiV strains. Further studies are needed to understand the disease implication of this new NiV strain on humans, along with the development of new diagnostics and treatment modalities.

## Supplementary Information

**Additional file 1: Supplementary Figure 1.** Alignment between the reference Nipah virus retrieved from human sequence of Bangladesh, India, 2004 (Accession Number: AY988601.1) and the bat samples of the Kerala, India, 2019. The figure was created in the CLC-genomics Workbench version 20.0.4. The genes encoded are marked in violet color and the green color display the proteins encoded by the reference NiV sequence. The quality scores are marked as the probability that ranges form 0–100% below each of the retrieved NiV sequences.

**Additional file 2: Supplementary Table 1.** Percentage nucleotide and amino acid similarity for each gene of Nipah virus with respect to the Nipah virus retrieved from human sequence of Bangladesh, India, 2004 (Accession Number: AY988601.1) outbreak.

**Additional file 3:** **Supplementary Table 2.** Mutation present in Indian NiV, 2019 outbreak compared to Bangladesh sequences.

**Additional file 4: Supplementary Table 3. **Nucleotide divergence for retrieved L gene sequences with respect to the L gene of of Nipah virus (Accession Number: AY988601.1).

## Data Availability

The data generated from this study is submitted to the public repository (GenBank).

## Abbreviations

NiV: Nipah virus
ICMR: Indian Council of Medical research
NIV: National Institute of Virology
qRTPCR: Real-time Reverse Transcriptase Polymerase Chain Reaction
ELISA: Enzyme-Linked Immuno-sorbent assay
BSL-4: Biosafety Level-4
NGS: Next Generation Sequencing

## References

[1] Chua KB (2003). Nipah virus outbreak in Malaysia. J Clin Virol.

[2] Chua KB, Bellini WJ, Rota PA (2000). Nipah virus: a recently emergent deadly paramyxovirus. Science.

[3] Mazzola LT, Kelly-Cirino C (2019). Diagnostics for Nipah virus: a zoonotic pathogen endemic to Southeast Asia. BMJ Glob Health.

[4] Sun B, Jia L, Liang B, Chen Q, Liu D (2018). Phylogeography, transmission, and viral proteins of Nipah virus. Virol Sin.

[5] Arankalle VA, Bandyopadhyay BT, Ramdasi AY (2011). Genomic characterization of Nipah virus, West Bengal, India. Emerg Infect Dis.

[6] Chadha MS, Comer JA, Lowe L (2006). Nipah virus-associated encephalitis outbreak, Siliguri, India. Emerg Infect Dis.

[7] Yadav P, Sudeep A, Gokhale M (2018). Circulation of Nipah virus in Pteropus giganteus bats in the northeast region of India, 2015. Indian J Med Res.

[8] Mourya DT, Yadav P, Sudeep AB (2019). Spatial association between a Nipah virus outbreak in India and Nipah virus infection in Pteropus bats. Clin Infect Dis.

[9] Guillaume V, Lefeuvre A, Faure C (2004). Specific detection of Nipah virus using real-time RT-PCR (TaqMan). J Virol Methods.

[10] Yadav P, Sudeep A, Gokhale M, Pawar S, Shete A, Patil D (2018). Circulation of Nipah virus in Pteropus giganteus bats in northeast region of India, 2015. Indian J Med Res.

[11] Yadav PD, Shete AM, Kumar GA (2019). Nipah virus sequences from humans and bats during Nipah outbreak, Kerala, India, 2018. Emerg Infect Dis.

[12] Yadav PD, Albariño CG, Nyayanit DA, Guerrero L, Jenks MH, Sarkale P (2018). Equine encephalosis virus in India, 2008. Emerg Infect Dis.

[13] Kumar S, Stecher G, Tamura K (2016). MEGA7: molecular evolutionary genetics analysis version 7.0 for bigger datasets. Mol Biol Evol.

[14] Mire CE, Geisbert JB, Agans KN (2019). Use of single-injection recombinant vesicular stomatitis virus vaccine to protect nonhuman primates against lethal Nipah virus disease. Emerg Infect Dis.

[15] Rahman SA, Hassan SS, Olival KJ (2010). Characterization of Nipah virus from naturally infected Pteropus vampyrus bats, Malaysia. Emerg Infect Dis.

[16] Kasloff SB, Leung A, Pickering BS (2019). Pathogenicity of Nipah henipavirus Bangladesh in a swine host. Sci Rep.

