# Supplementary figures and images for: Detection of Nipah virus in Pteropus medius in 2019 outbreak from Ernakulam district, Kerala, India
Source: BMC Infect Dis. 2021 Feb 9;21:162. doi: 10.1186/s12879-021-05865-7 (PMC7871573; doi:10.1186/s12879-021-05865-7)

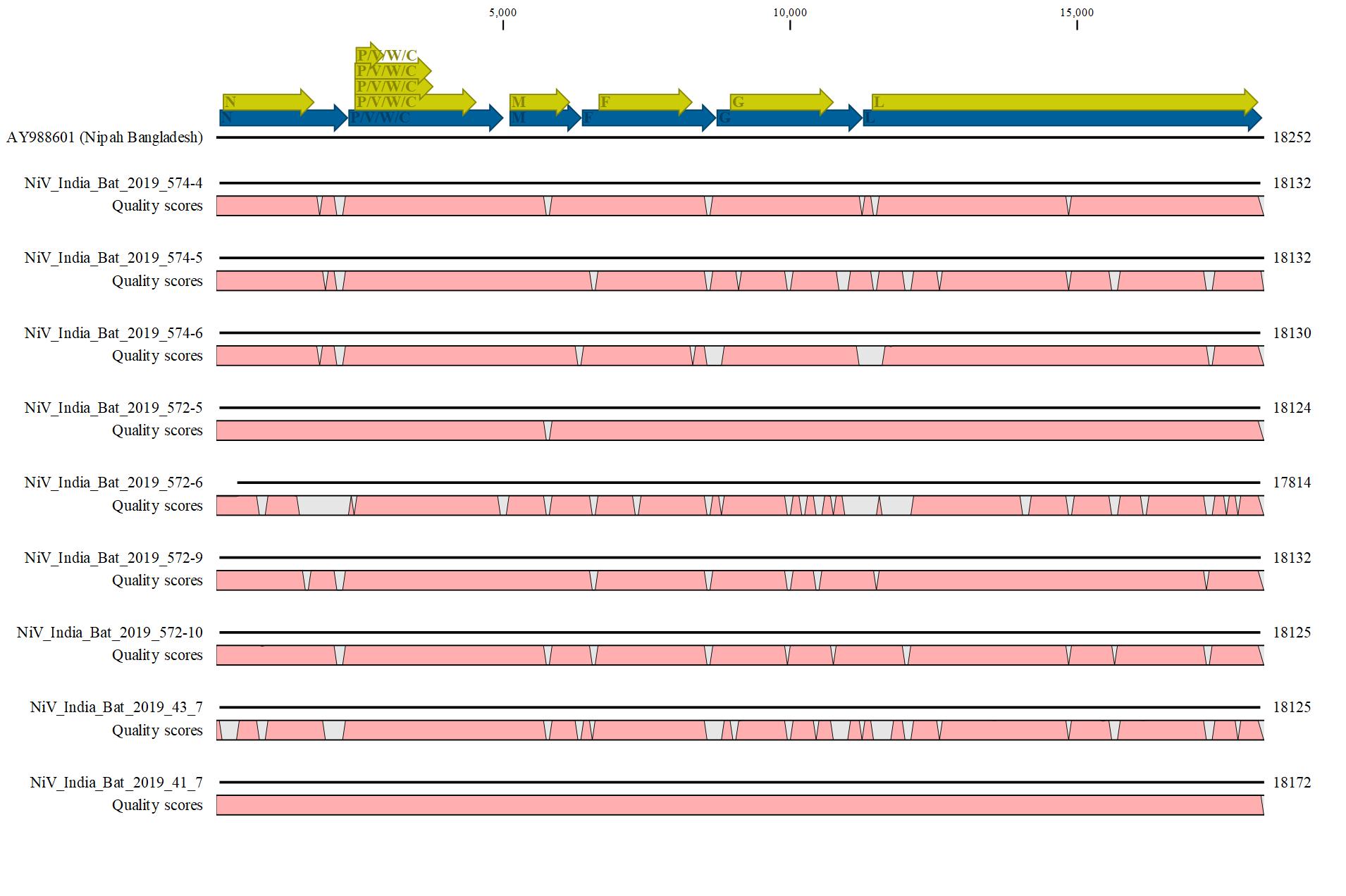

Supplement: Supplementary file 1 — Additional file 1: Supplementary Figure 1. Alignment between the reference Nipah virus retrieved from human sequence of Bangladesh, India, 2004 (Accession Number: AY988601.1) and the bat samples of the Kerala, India, 2019. The figure was created in the CLC-genomics Workbench version 20.0.4. The genes encoded are marked in violet color and the green color display the proteins encoded by the reference NiV sequence. The quality scores are marked as the probability that ranges form 0–100% below each of the retrieved NiV sequences. [file 12879_2021_5865_MOESM1_ESM.jpg]
