# Supplementary material for: Detection of Nipah virus in Pteropus medius in 2019 outbreak from Ernakulam district, Kerala, India
Source: BMC Infect Dis. 2021 Feb 9;21:162. doi: 10.1186/s12879-021-05865-7 (PMC7871573; doi:10.1186/s12879-021-05865-7)
Supplement: Supplementary file 2 — Additional file 2: Supplementary Table 1. Percentage nucleotide and amino acid similarity for each gene of Nipah virus with respect to the Nipah virus retrieved from human sequence of Bangladesh, India, 2004 (Accession Number: AY988601.1) outbreak. [file 12879_2021_5865_MOESM2_ESM.docx]

Supplementary Table 1: Percentage nucleotide and amino acid similarity for each gene of Nipah virus with respect to the Nipah virus retrieved from human sequence of Bangladesh, India, 2004 (Accession Number: AY988601.1) outbreak.

|  | **N** | | **P** | |  | **M** | | **F** | | **G** | | **L** | |
| --- | --- | --- | --- | --- | --- | --- | --- | --- | --- | --- | --- | --- | --- |
|  | **PNS** | **PAS** | **PNS** | **PAS** |  | **PNS** | **PAS** | **PNS** | **PAS** | **PNS** | **PAS** | **PNS** | **PAS** |
| MK575067.1_NiV/BD/P.medius/EHA/2013/Raypur1409 | 99.7 | 99.8 | 99.2 | 99.2 |  | 100.0 | 100.0 | 99.5 | 99.6 | 99.8 | 99.8 | 99.7 | 99.9 |
| MK575061.1_NiV/BD/P.medius/EHA/2013/Raypur1402 | 99.7 | 99.8 | 99.2 | 99.3 |  | 100.0 | 100.0 | 99.5 | 99.6 | 99.8 | 99.8 | 99.7 | 99.9 |
| MK575062.1_NiV/BD/P.medius/EHA/2013/Raypur1403 | 99.7 | 99.8 | 99.2 | 99.3 |  | 100.0 | 100.0 | 99.5 | 99.6 | 99.8 | 99.8 | 99.7 | 99.9 |
| MK575064.1_NiV/BD/P.medius/EHA/2013/Raypur1405 | 99.7 | 99.8 | 99.2 | 99.3 |  | 100.0 | 100.0 | 99.5 | 99.6 | 99.8 | 99.8 | 99.7 | 99.9 |
| MK575068.1_NiV/BD/P.medius/EHA/2013/Raypur1410 | 99.7 | 99.8 | 99.2 | 99.2 |  | 100.0 | 100.0 | 99.5 | 99.6 | 99.8 | 99.8 | 99.7 | 99.9 |
| MK575065.1_NiV/BD/P.medius/EHA/2013/Raypur1406 | 99.7 | 99.8 | 99.2 | 99.3 |  | 100.0 | 100.0 | 99.5 | 99.6 | 99.8 | 99.8 | 99.7 | 99.9 |
| MK575069.1_NiV/BD/P.medius/EHA/2013/Raypur1411 | 99.7 | 99.8 | 99.2 | 99.3 |  | 100.0 | 100.0 | 99.5 | 99.6 | 99.7 | 99.8 | 99.7 | 99.8 |
| MK575063.1_NiV/BD/P.medius/EHA/2013/Raypur1404 | 99.7 | 99.8 | 99.2 | 99.3 |  | 100.0 | 100.0 | 99.5 | 99.6 | 99.7 | 99.8 | 99.7 | 99.8 |
| MK575070.1_NiV/BD/P.medius/EHA/2013/Sylhet191 | 99.6 | 99.8 | 99.2 | 99.3 |  | 100.0 | 100.0 | 99.5 | 99.6 | 99.7 | 99.8 | 99.6 | 99.7 |
| MK575066.1_NiV/BD/P.medius/EHA/2013/Raypur1408 | 99.7 | 99.8 | 99.2 | 99.3 |  | 100.0 | 100.0 | 99.5 | 99.6 | 99.8 | 99.8 | 99.7 | 99.9 |
| JN808864.1_NIV/BD/2010/FARIDPUR | 99.7 | 99.6 | 99.6 | 99.7 |  | 100.0 | 100.0 | 99.5 | 99.6 | 99.8 | 99.8 | 99.8 | 99.9 |
| MK575060.1_NiV/BD/P.medius/EHA/2013/Raypur1401 | 99.7 | 99.8 | 99.2 | 99.3 |  | 100.0 | 100.0 | 99.5 | 99.6 | 99.8 | 99.8 | 99.7 | 99.8 |
| JN808857.1_NIV/BD/2008/MANIKGONJ | 99.4 | 99.8 | 99.3 | 99.3 |  | 99.8 | 100.0 | 99.0 | 99.5 | 99.6 | 100.0 | 99.2 | 99.7 |
| JN808863.1_NIV/BD/2008/RAJBARI | 99.4 | 99.8 | 99.3 | 99.3 |  | 99.8 | 100.0 | 99.0 | 99.5 | 99.6 | 100.0 | 99.2 | 99.7 |
| FJ513078.1_NiV/India/HU/2007/FG | 99.4 | 99.6 | 99.5 | 99.4 |  | 99.7 | 99.7 | 99.3 | 99.5 | 99.6 | 99.8 | 99.3 | 99.7 |
| MH396625.1_NiV/India/HU/2018 | 98.7 | 99.2 | 98.2 | 98.2 |  | 98.9 | 100.0 | 98.4 | 99.5 | 98.2 | 99.0 | 98.2 | 99.7 |
| MH523641.1_NiV/India/HU/2018 | 98.7 | 99.2 | 98.2 | 98.2 |  | 98.9 | 100.0 | 98.4 | 99.5 | 98.2 | 99.0 | 98.2 | 99.7 |
| MH523640.1_NiV/India/HU/2018 | 98.7 | 99.2 | 98.2 | 98.2 |  | 98.9 | 100.0 | 98.4 | 99.5 | 98.2 | 99.0 | 98.2 | 99.7 |
| MH523642.1_NiV/India/HU/2018 | 98.7 | 99.2 | 98.2 | 98.0 |  | 98.9 | 100.0 | 98.4 | 99.5 | 98.2 | 99.0 | 98.2 | 99.7 |
| NiV/India/Bat/2019/572-9 | 98.9 | 99.4 | 98.3 | 98.3 |  | 99.0 | 100.0 | 98.2 | 99.5 | 97.9 | 98.5 | 98.1 | 99.6 |
| NiV/India/Bat/2019/572-5 | 98.7 | 99.1 | 98.3 | 98.4 |  | 98.9 | 100.0 | 98.2 | 99.5 | 97.9 | 98.7 | 98.1 | 99.6 |
| NiV/India/Bat/2019/41/7 | 98.7 | 99.1 | 98.3 | 98.4 |  | 99.0 | 100.0 | 98.2 | 99.5 | 97.9 | 98.7 | 98.1 | 99.6 |
| NiV/India/Bat/2019/574-4 | 98.5 | 99.1 | 98.1 | 98.2 |  | 99.0 | 100.0 | 98.0 | 99.1 | 98.1 | 98.8 | 98.2 | 99.7 |
| NiV/India/Bat/2019/572-10 | 97.8 | 97.7 | 98.3 | 98.3 |  | 98.9 | 100.0 | 98.2 | 99.5 | 97.9 | 98.8 | 98.0 | 99.6 |
| NiV/India/Bat/2019/574-6 | 98.6 | 99.2 | 98.2 | 98.3 |  | 99.0 | 100.0 | 98.1 | 99.1 | 98.2 | 99.0 | 98.1 | 99.5 |
| NiV/India/Bat/2019/574-5 | 98.6 | 99.2 | 98.1 | 98.2 |  | 98.8 | 99.7 | 98.0 | 99.1 | 98.0 | 99.1 | 98.1 | 99.6 |
| NiV/India/Bat/2019/43/7 | 98.1 | 97.3 | 98.2 | 98.2 |  | 98.8 | 99.7 | 98.1 | 99.5 | 97.6 | 97.9 | 97.7 | 98.8 |
| NiV/India/Bat/2019/572-6 | 98.2 | 97.7 | 98.2 | 98.4 |  | 98.0 | 99.0 | 98.4 | 99.6 | 97.7 | 98.5 | 97.9 | 99.4 |
| AY029768.1_NiV/MY/HU/1999/UMMC2 | 94.3 | 98.3 | 92.0 | 91.9 |  | 93.4 | 98.9 | 93.5 | 98.5 | 93.0 | 95.7 | 93.4 | 98.3 |
| KY425655.1_NiV/MY/HU/IRF0158 | 94.3 | 98.3 | 92.0 | 91.9 |  | 93.4 | 98.9 | 93.5 | 98.5 | 93.0 | 95.7 | 93.5 | 98.3 |
| AJ564622.1_NiV/MY/Pig/99/VRI-1413 | 94.3 | 98.3 | 92.0 | 91.9 |  | 93.4 | 98.9 | 93.5 | 98.5 | 93.0 | 95.7 | 93.5 | 98.3 |
| KY425646.1_NiV/MY/HU/1999/IRF0160 | 94.3 | 98.3 | 92.0 | 91.9 |  | 93.4 | 98.9 | 93.5 | 98.5 | 93.0 | 95.7 | 93.5 | 98.3 |
| AJ564623.1_NiV/MY/Pig/99/UM-0128 | 94.3 | 98.3 | 92.0 | 91.9 |  | 93.4 | 98.9 | 93.5 | 98.5 | 93.0 | 95.7 | 93.4 | 98.3 |
| AY029767.1_NiV/MY/HU/1999/UMMC1 | 94.3 | 98.3 | 92.0 | 91.9 |  | 93.4 | 98.9 | 93.5 | 98.5 | 93.0 | 95.7 | 93.5 | 98.3 |
| NC_002728.1_NiV/MY/HU/1999/CDC | 94.3 | 98.3 | 92.0 | 91.9 |  | 93.4 | 98.9 | 93.5 | 98.5 | 93.0 | 95.7 | 93.5 | 98.3 |
| AJ564621.1_NV/MY/Pig/99/VRI-2794 | 94.3 | 98.3 | 92.0 | 91.9 |  | 93.4 | 98.9 | 93.4 | 98.5 | 93.0 | 95.7 | 93.5 | 98.3 |
| AJ627196.1_NV/MY/Pig/99/VRI-0626 | 94.1 | 97.9 | 91.9 | 91.8 |  | 93.5 | 99.1 | 93.4 | 98.4 | 93.0 | 95.7 | 93.4 | 98.1 |
| FN869553_NiV/MY/Bat/2008/Perek | 94.4 | 98.9 | 91.3 | 90.4 |  | 93.8 | 99.1 | 93.5 | 98.3 | 93.3 | 96.0 | 93.4 | 98.1 |
| AF376747.1_NiV/MY/Pig/1999/VRI-0626 | 94.2 | 98.1 | 91.9 | 91.8 |  | 93.4 | 98.9 | 93.4 | 98.4 | 92.9 | 95.7 | NA | NA |

**Foot notes:**

**Color code: Black- Bangladesh sequences; Blue –Indian sequences; Purple – Malaysian sequences.**
